# Metabolic-vascular burden and subsequent Parkinson’s disease across Chinese, English, and European ageing cohorts

**DOI:** 10.21203/rs.3.rs-10123159/v1

**Published:** 2026-07-07

**Authors:** Shan Zeng, Aishanjiang Yusufujiang, Hongyan Li

**Affiliations:** People’s Hospital of Xinjiang Uygur Autonomous Region; People’s Hospital of Xinjiang Uygur Autonomous Region; People’s Hospital of Xinjiang Uygur Autonomous Region

**Keywords:** Parkinson’s disease, metabolic syndrome, diabetes, hypertension, obesity, ageing cohorts, cohort study

## Abstract

**Background:**

Metabolic and vascular disorders have been linked to Parkinson’s disease (PD), but population-based evidence remains inconsistent. We examined whether harmonized metabolic-vascular burden was associated with subsequent reported PD across Chinese, English, and European ageing cohorts.

**Methods:**

We analyzed participants aged 50 years or older in CHARLS, ELSA, and SHARE after excluding reported PD at exposure baseline. Metabolic-vascular burden was scored from 0 to 4 by summing diabetes, hypertension, dyslipidemia or high cholesterol, and body mass index > = 25 kg/m2. Subsequent reported PD was identified at follow-up waves. Cohort-specific logistic regression estimated odds ratios (ORs) per additional component, followed by random-effects meta-analysis.

**Results:**

The analytic sample included 111,142 participants and 1,332 follow-up PD events: 16,531 participants and 241 events in CHARLS, 13,581 and 134 in ELSA, and 81,030 and 957 in SHARE. In demographic-adjusted models, each additional component was associated with higher PD odds in CHARLS (OR 1.29, 95% CI 1.14–1.47) and SHARE (OR 1.13, 95% CI 1.07–1.20), but not ELSA (OR 1.03, 95% CI 0.85–1.25). The pooled demographic-adjusted OR was 1.16 (95% CI 1.04–1.29; I2 = 59.5%). Fully adjusted estimates were attenuated (pooled OR 1.07, 95% CI 0.90–1.28). Component models implicated diabetes and dyslipidemia in CHARLS and diabetes and BMI > = 25 kg/m2 in SHARE.

**Conclusions:**

Metabolic-vascular burden showed a modest, heterogeneous association with subsequent reported PD. The findings support interpreting cardiometabolic-PD associations as population-context dependent rather than as a single transportable risk estimate.

## 1. Introduction

Parkinson’s disease (PD) is a common neurodegenerative disorder characterized by motor, non-motor, autonomic, cognitive, and neuropsychiatric manifestations. Its public health importance is increasing as populations age and as survival with chronic disease improves [1, 2, 15, 16]. Although age, sex, genetic susceptibility, environmental exposures, prodromal symptoms, and lifestyle factors contribute to PD epidemiology, modifiable cardiometabolic conditions have attracted growing interest because they are common, clinically measurable, and biologically plausible contributors to neurodegenerative vulnerability [3–9, 17–19].

Diabetes, adiposity, dyslipidemia, and hypertension may influence PD-related processes through overlapping inflammatory, vascular, mitochondrial, oxidative-stress, insulin-signaling, and blood-brain-barrier pathways [3–7, 20]. Diabetes has been associated with higher PD risk and more severe disease in several systematic reviews and population studies [3, 4, 8, 21]. Metabolic syndrome has also been associated with incident PD in large administrative and cohort datasets [5]. However, the individual components do not show uniform associations. Hypertension, body mass index (BMI), and lipid measures have shown positive, null, or inverse associations across studies, depending on the cohort, age at exposure measurement, medication use, follow-up duration, competing mortality, and the timing of risk-factor assessment relative to prodromal PD [6, 7, 9, 22, 23, 25]. These inconsistencies make it difficult to infer whether metabolic-vascular risk should be treated as a generalizable PD risk marker or as a context-sensitive signal.

Cross-national ageing studies offer a useful design for addressing this problem. The China Health and Retirement Longitudinal Study (CHARLS), English Longitudinal Study of Ageing (ELSA), and Survey of Health, Ageing and Retirement in Europe (SHARE) were designed to support harmonized analyses of health, function, and socioeconomic conditions in middle-aged and older adults [10–13]. They include repeated assessments of physician-diagnosed chronic diseases and anthropometric or self-reported health measures, making it possible to construct a pragmatic metabolic-vascular burden score across populations. Such a score cannot replace clinical metabolic syndrome definitions, but it captures the cumulative burden of common cardiometabolic conditions available in comparable form across cohorts.

We therefore examined the association between metabolic-vascular burden and subsequent reported PD in CHARLS, ELSA, and SHARE. Our primary aim was to estimate cohort-specific and pooled associations per additional metabolic-vascular component. Our secondary aims were to evaluate cross-cohort heterogeneity, determine whether individual components drove the observed associations, and test the robustness of findings in older participants and after excluding PD reports occurring soon after exposure baseline. We hypothesized that higher metabolic-vascular burden would be associated with higher subsequent PD odds, but that the magnitude of association would vary by cohort.

## 2. Methods

### 2.1. Study Design And Reporting

This was a longitudinal observational cohort analysis of harmonized ageing data from CHARLS, ELSA, and SHARE. The exposure baseline was defined separately for each participant as the first wave at which sufficient metabolic-vascular information was available. Participants were then followed through subsequent waves for reported PD. The analysis was designed to preserve temporal ordering by excluding participants with reported PD at exposure baseline and by defining the outcome from later waves only. The manuscript follows the Strengthening the Reporting of Observational Studies in Epidemiology (STROBE) guidance for cohort studies [14].

### 2.2. Data Sources

CHARLS is a nationally representative longitudinal survey of middle-aged and older adults in China [10]. ELSA is a longitudinal study of adults aged 50 years or older in England [12]. SHARE is a multicountry longitudinal survey of health, ageing, and retirement in Europe [11]. Harmonized variables were derived through the Gateway to Global Aging Data framework, which supports cross-national comparisons while retaining cohort-specific measurement characteristics [13]. The present analysis used deidentified secondary data and did not involve new participant contact.

### 2.3. Study Population

Participants were eligible if they were aged 50 years or older at exposure baseline, had no reported PD at that baseline, had at least one subsequent PD assessment, and had at least three nonmissing metabolic-vascular components. The final analytic samples were 16,531 participants in CHARLS, 13,581 in ELSA, and 81,030 in SHARE. Mean follow-up was summarized by the number of available follow-up waves rather than person-years because survey timing and wave spacing differed across cohorts. The mean number of follow-up waves was 3.13 in CHARLS, 4.50 in ELSA, and 3.24 in SHARE.

### 2.4. Exposure Definition

The primary exposure was a composite metabolic-vascular burden score ranging from 0 to 4. One point was assigned for each of the following components: diabetes, hypertension, dyslipidemia or high cholesterol, and BMI > = 25 kg/m2. Diabetes, hypertension, and dyslipidemia or high cholesterol were based on harmonized reports of physician diagnosis or cohort-available disease indicators. BMI was derived from available height and weight information when present and was dichotomized at 25 kg/m2 to provide a common threshold across cohorts. Participants with at least three observed components were retained; the score reflected the sum of observed positive components. For descriptive analyses, scores were grouped as 0, 1, 2, and 3–4 because the highest scores had smaller cell counts in some cohorts.

The score was intended as a pragmatic cumulative burden measure rather than a formal metabolic syndrome diagnosis. This approach was chosen because waist circumference, triglycerides, high-density lipoprotein cholesterol, fasting glucose, measured blood pressure, and medication indicators required for standard metabolic syndrome definitions were not uniformly available across all cohorts and waves [24]. The harmonized burden score therefore prioritizes cross-cohort comparability and interpretability.

### 2.5. Outcome Definition

The outcome was subsequent reported PD during follow-up. Participants were classified as having a follow-up PD event if PD was reported at any wave after exposure baseline. Participants with reported PD at exposure baseline were excluded to reduce reverse temporal ordering. Because PD ascertainment relied on survey-reported physician diagnosis or harmonized disease indicators rather than adjudicated clinical examination, the outcome should be interpreted as reported PD.

### 2.6. Covariates

Covariates were selected to support comparable adjustment across cohorts while avoiding adjustment for variables unavailable in one or more datasets. Demographic-adjusted models included age, sex, and education. Fully adjusted models additionally included smoking, drinking, physical activity, and cardiometabolic variables as available in the analytic data. Because some covariates overlapped conceptually with the exposure score, the fully adjusted models were interpreted as conservative attenuation analyses rather than as the primary etiologic specification. Complete-case sample sizes were lower in fully adjusted analyses, especially in CHARLS and ELSA, and this loss of precision was considered when interpreting results.

### 2.7. Statistical Analysis

Baseline characteristics were summarized by cohort and metabolic-vascular burden group. Continuous variables are presented as mean (standard deviation), and categorical variables as number (percentage). Follow-up PD occurrence was summarized within burden categories to describe absolute risk patterns.

The primary analysis used cohort-specific logistic regression models to estimate ORs and 95% confidence intervals (CIs) for subsequent reported PD per one additional metabolic-vascular component. Three model specifications were fitted: unadjusted, demographic-adjusted, and fully adjusted. The demographic-adjusted model was prespecified as the primary model because it offered a consistent adjustment set across cohorts and preserved the exposure as a cumulative burden construct.

To summarize evidence across cohorts, we pooled cohort-specific log ORs using random-effects meta-analysis [26]. Heterogeneity was described using I2. We emphasized random-effects estimates because the cohorts differed in geography, health-care systems, age structure, chronic disease measurement, and PD ascertainment. Fixed universal effects were not assumed.

Secondary component analyses fitted mutually adjusted models including diabetes, hypertension, dyslipidemia or high cholesterol, and BMI > = 25 kg/m2 as separate predictors. These analyses were used to identify which components contributed most strongly to the burden association within each cohort. Prespecified sensitivity analyses repeated the burden model among participants aged 60 years or older and after excluding PD events reported within one follow-up wave of exposure baseline. The older-age analysis assessed whether associations were robust in the age range with higher PD incidence. The early-event exclusion analysis reduced the influence of undiagnosed or prodromal PD already present near baseline.

All analyses were conducted separately by cohort before meta-analysis. Statistical significance was evaluated using two-sided *p* values, with emphasis on effect sizes, confidence intervals, and heterogeneity rather than on isolated threshold testing.

## 3. Results

### 3.1. Analytic Sample And Baseline Profile

**Figure 1:**
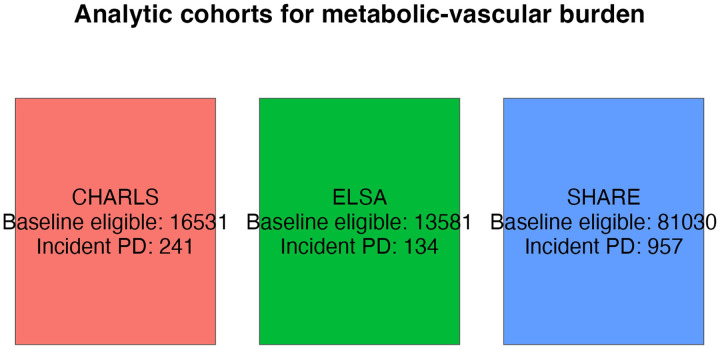
Study flow for the metabolic-vascular burden analytic cohorts. The figure summarizes the three cohort-specific analytic samples used to evaluate metabolic-vascular burden and subsequent reported Parkinson’s disease. Participants were eligible if they were aged 50 years or older, had no reported Parkinson’s disease at exposure baseline, had sufficient metabolic-vascular data, and had at least one subsequent Parkinson’s disease assessment.

The study included 111,142 participants and 1,332 follow-up reported PD events across the three cohorts. CHARLS contributed 16,531 participants and 241 events, ELSA contributed 13,581 participants and 134 events, and SHARE contributed 81,030 participants and 957 events (Fig. 1; Supplementary Table 2). The larger SHARE sample provided most events, whereas CHARLS showed the steepest event gradient across metabolic-vascular burden categories.

Baseline characteristics varied substantially by cohort and burden group (Table 1). In CHARLS, follow-up PD occurrence was 1.1% among participants with burden score 0, 1.6% with score 1, 2.4% with score 2, and 2.1% with score 3–4. Mean age increased only modestly across CHARLS burden groups, from 58.0 years at score 0 to 58.8 years at score 3–4. In ELSA, PD occurrence ranged from 0.7% at score 0 to 1.0% at score 3–4, with older age at higher burden levels. Mean age increased from 59.4 years at score 0 to 65.6 years at score 3–4. In SHARE, PD occurrence increased from 0.9% at score 0 to 1.7% at score 3–4, and mean age increased from 61.8 to 66.1 years across the same contrast.

The composition of the burden score differed by cohort. BMI > = 25 kg/m2 was common among participants with any metabolic-vascular burden, particularly in ELSA and SHARE. Hypertension became highly prevalent in the score 2 and score 3–4 groups in all cohorts. Diabetes and dyslipidemia or high cholesterol were concentrated in the highest burden category. These descriptive patterns indicate that the same numeric burden score did not necessarily represent identical clinical profiles across populations.

### 3.2. Metabolic-Vascular Burden And Subsequent Reported PD

**Figure 3:**
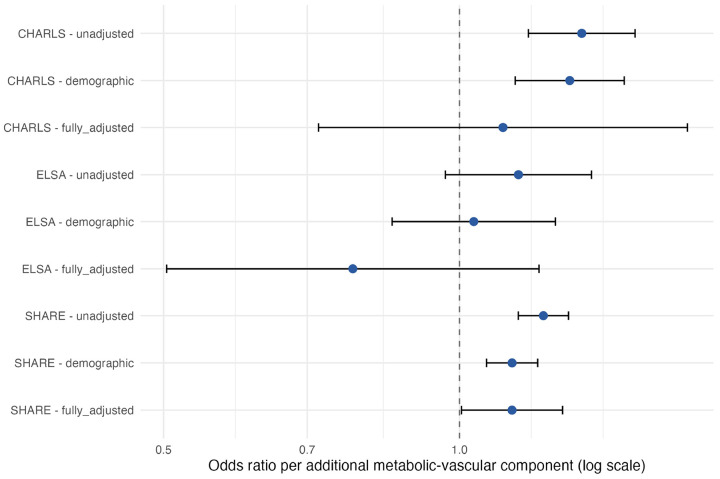
Cohort-specific associations between metabolic-vascular burden and subsequent reported Parkinson’s disease. The forest plot shows odds ratios and 95% confidence intervals from unadjusted, demographic-adjusted, and fully adjusted logistic regression models. Odds ratios are expressed per one additional metabolic-vascular component. The vertical reference line indicates an odds ratio of 1.

In unadjusted cohort-specific models, each additional metabolic-vascular component was associated with higher odds of subsequent reported PD in CHARLS (OR 1.33, 95% CI 1.18–1.51; *p* < 0.001) and SHARE (OR 1.22, 95% CI 1.15–1.29; *p* < 0.001), whereas the ELSA estimate was weaker and not statistically significant (OR 1.15, 95% CI 0.97–1.36; *p* = 0.114) (Table 2; Fig. 3).

After adjustment for age, sex, and education, the association remained evident in CHARLS and SHARE. In CHARLS, each additional component was associated with 29% higher odds of subsequent reported PD (OR 1.29, 95% CI 1.14–1.47; *p* < 0.001). In SHARE, the corresponding estimate was smaller but precise (OR 1.13, 95% CI 1.07–1.20; *p* < 0.001). In ELSA, the demographic-adjusted estimate was close to the null (OR 1.03, 95% CI 0.85–1.25; *p* = 0.732).

Random-effects pooling of the demographic-adjusted estimates produced a pooled OR of 1.16 (95% CI 1.04–1.29; *p* = 0.007), with moderate heterogeneity (I2 = 59.5%) (Table 3). The unadjusted pooled OR was 1.23 (95% CI 1.17–1.29; *p* < 0.001) and showed minimal heterogeneity (I2 = 0.1%), suggesting that differences in age, sex, education, and cohort structure contributed to the emergence of between-cohort variation after adjustment.

Fully adjusted models showed attenuation and reduced precision. The CHARLS fully adjusted model included 4,474 participants and 73 events and yielded an OR of 1.11 (95% CI 0.72–1.71; *p* = 0.644). The ELSA fully adjusted model included 7,418 participants and 83 events and yielded an OR of 0.78 (95% CI 0.50–1.20; *p* = 0.261). The SHARE fully adjusted model retained 78,283 participants and 918 events and yielded an OR of 1.13 (95% CI 1.00–1.27; *p* = 0.041). The pooled fully adjusted OR was 1.07 (95% CI 0.90–1.28; *p* = 0.455; I2 = 22.1%). Because full adjustment introduced overlapping cardiometabolic covariates and larger complete-case exclusions, these results were interpreted primarily as evidence that the association was not robust to conservative adjustment and missingness.

### 3.3. Component Analyses

**Figure 4:**
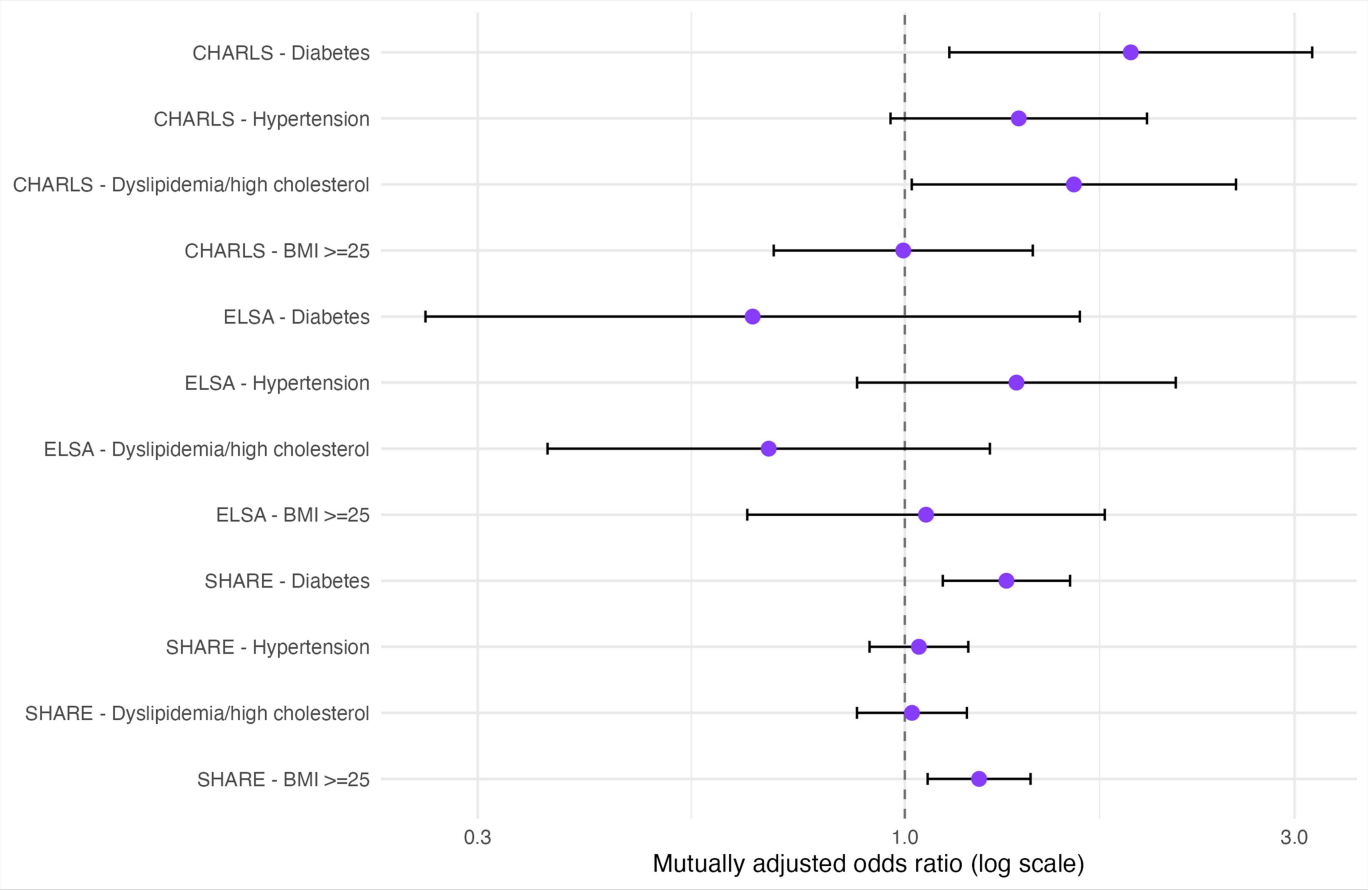
Mutually adjusted component associations with subsequent reported Parkinson’s disease. The forest plot shows cohort-specific odds ratios and 95% confidence intervals for diabetes, hypertension, dyslipidemia or high cholesterol, and BMI >=25 kg/m2 in mutually adjusted component models. Estimates identify which components contributed most strongly to the overall burden association in each cohort.

Mutually adjusted component models suggested that different components contributed to the burden association in different cohorts (Table 4; Fig. 4). In CHARLS, diabetes was associated with higher subsequent reported PD odds (OR 1.89, 95% CI 1.13–3.15; *p* = 0.015), as was dyslipidemia or high cholesterol (OR 1.61, 95% CI 1.02–2.54; *p* = 0.041). Hypertension showed a positive but imprecise association (OR 1.38, 95% CI 0.96–1.98; *p* = 0.082), whereas BMI > = 25 kg/m2 was not associated with PD (OR 1.00, 95% CI 0.69–1.43; *p* = 0.981).

In ELSA, none of the individual components showed a statistically clear association. Diabetes and dyslipidemia or high cholesterol had point estimates below 1, hypertension had a positive but imprecise estimate, and BMI > = 25 kg/m2 was close to the null. The wide CIs reflect the smaller number of PD events available for component modeling.

In SHARE, diabetes and BMI > = 25 kg/m2 were the clearest contributors. Diabetes was associated with higher PD odds (OR 1.33, 95% CI 1.11–1.59; *p* = 0.002), and BMI > = 25 kg/m2 was also associated with higher odds (OR 1.23, 95% CI 1.07–1.43; *p* = 0.005). Hypertension and dyslipidemia or high cholesterol were close to the null in SHARE. Thus, the overall burden score combined partly different component signals across cohorts.

### 3.4. Sensitivity Analyses

Sensitivity analyses supported the main pattern in CHARLS and SHARE but not ELSA (Table 5; Supplementary Fig. 1). Among participants aged 60 years or older, each additional metabolic-vascular component was associated with higher PD odds in CHARLS (OR 1.34, 95% CI 1.13–1.57; *p* < 0.001) and SHARE (OR 1.10, 95% CI 1.04–1.18; *p* = 0.003). The corresponding ELSA estimate remained null (OR 0.97, 95% CI 0.78–1.21; *p* = 0.799).

After excluding PD events reported within one follow-up wave of exposure baseline, associations also persisted in CHARLS (OR 1.27, 95% CI 1.11–1.46; *p* < 0.001) and SHARE (OR 1.12, 95% CI 1.05–1.21; *p* < 0.001), but not ELSA (OR 1.05, 95% CI 0.85–1.31; *p* = 0.623). These findings reduce, but do not eliminate, concern that the primary associations in CHARLS and SHARE were driven solely by PD already present but unreported at baseline.

## 4. Discussion

### 4.1. Principal Findings

In this cross-cohort analysis of 111,142 middle-aged and older adults, higher metabolic-vascular burden was associated with subsequent reported PD in CHARLS and SHARE, but not in ELSA. The pooled demographic-adjusted estimate suggested a modest association: each additional metabolic-vascular component corresponded to 16% higher odds of subsequent reported PD. However, moderate heterogeneity and attenuation in fully adjusted models indicate that the association should not be interpreted as a single universal effect.

The burden score also appeared to represent different clinical patterns across populations. In CHARLS, diabetes and dyslipidemia or high cholesterol were the strongest component signals. In SHARE, diabetes and BMI > = 25 kg/m2 were more prominent. In ELSA, no component showed a clear association. These differences are central to the interpretation of the study: the same cumulative score may index different combinations of disease biology, treatment exposure, detection patterns, and survival selection across cohorts.

### 4.2. Comparison With Previous Evidence

Our results align partly with prior studies linking diabetes and metabolic syndrome to PD risk [3–5, 8, 21]. The positive associations for diabetes in CHARLS and SHARE are consistent with literature suggesting that insulin resistance, systemic inflammation, mitochondrial dysfunction, and vascular injury may contribute to neurodegenerative susceptibility [3, 4, 8, 20, 21]. The SHARE association for BMI > = 25 kg/m2 is also compatible with evidence that adiposity-related metabolic dysfunction may be relevant to PD risk in some populations, although prior BMI findings have been heterogeneous and potentially affected by survival and reverse-causation mechanisms [7, 22]. The dyslipidemia result in CHARLS should also be interpreted cautiously because lipid-PD studies have yielded mixed findings across prospective and case-control settings [23]. Overall, the lack of a clear ELSA association and the null or inconsistent component estimates for hypertension and dyslipidemia show why single-component or single-cohort results may not generalize cleanly.

Several explanations may account for the heterogeneity. First, the cohorts differ in age distribution, cardiometabolic treatment patterns, health-care access, and diagnostic behavior. Treated hypertension or dyslipidemia may carry different implications from untreated disease, but medication intensity and duration were not harmonized in the primary exposure. Second, PD ascertainment depended on reported diagnosis. Diagnosis may be delayed or underreported in settings with less specialist access, and awareness may vary by education, cohort wave, and health system. Third, survival and competing-risk processes may differ. Individuals with severe cardiometabolic disease may be less likely to survive to an age at which PD is diagnosed, potentially attenuating associations in older or heavily treated populations. Fourth, prodromal PD can affect weight, autonomic function, physical activity, and smoking behavior before diagnosis, complicating exposure timing. Excluding early follow-up events helped address this issue but cannot fully remove prodromal influence.

The attenuation after full adjustment also requires careful interpretation. Adjustment for smoking, drinking, physical activity, and individual cardiometabolic variables may reduce confounding, but it may also adjust away parts of the cumulative burden construct or introduce missing-data selection. In CHARLS, the fully adjusted complete-case sample was much smaller than the demographic-adjusted sample, limiting precision. For this reason, the demographic-adjusted model is the most comparable cross-cohort specification, whereas the fully adjusted model is best viewed as a conservative robustness analysis.

### 4.3. Implications

These findings have methodological and clinical implications. Methodologically, they support analyzing metabolic-PD associations in a framework that explicitly tests heterogeneity rather than assuming transportability across populations. A pooled estimate can be useful, but it should be presented alongside cohort-specific estimates and differences in component composition. Clinically and epidemiologically, the results suggest that cardiometabolic burden may help identify groups with elevated reported PD occurrence in some settings, but it is not a stand-alone screening marker and should not be interpreted without population context.

The results also suggest priorities for future research. Studies with adjudicated PD outcomes, biomarker data, medication histories, genetic susceptibility, and repeated cardiometabolic measurements could clarify whether observed heterogeneity reflects biological effect modification, differential diagnosis, treatment patterns, or selection. Harmonized cohorts that include both Asian and European populations are particularly valuable because they allow the same analytic question to be tested under different demographic and health-system conditions.

### 4.4. Strengths And Limitations

This study has several strengths. It used three large ageing cohorts with harmonized variables, applied a longitudinal exposure-outcome ordering, excluded participants with reported PD at baseline, and evaluated both cohort-specific and pooled estimates. The analysis included more than 111,000 participants and 1,332 follow-up reported PD events. It also examined individual metabolic-vascular components and performed sensitivity analyses in older participants and after excluding early follow-up PD reports.

The study also has important limitations. PD was based on reported diagnosis rather than clinical adjudication, which may lead to under-ascertainment or misclassification. The metabolic-vascular burden score used pragmatic harmonized indicators and did not implement formal metabolic syndrome criteria. BMI > = 25 kg/m2 may have different cardiometabolic meanings across ethnic groups and age ranges, and the score did not incorporate waist circumference or laboratory values uniformly across cohorts. Exposure status was measured at an exposure baseline and may not capture duration, severity, treatment, or changes over follow-up. Fully adjusted models were limited by missingness and by overlap between adjustment variables and the exposure construct. Logistic regression summarized subsequent PD occurrence but did not model exact event times or competing mortality. Finally, residual confounding by physical function, prodromal symptoms, medications, socioeconomic conditions, environmental exposures, and genetic susceptibility remains possible.

## 5. Conclusion

Metabolic-vascular burden was modestly associated with subsequent reported PD across harmonized ageing cohorts, but the association was heterogeneous. CHARLS and SHARE showed positive demographic-adjusted associations that persisted in sensitivity analyses, whereas ELSA did not. Component analyses suggested that diabetes, dyslipidemia or high cholesterol, and BMI > = 25 kg/m2 contributed differently across cohorts. These findings support a population-specific interpretation of cardiometabolic-PD associations and highlight the need for adjudicated, biomarker-enhanced, and treatment-aware longitudinal studies.

## Supplementary Material

Supplementary Files

This is a list of supplementary files associated with this preprint. Click to download.

- Table1baselinecharacteristics.csv
- Table4componentmodels.csv
- Table2primarymodels.csv
- Table5sensitivityanalyses.csv
- SupplementaryFigure1sensitivityforest.png
- Table3metaanalysis.csv
- SupplementaryTable2sampleflow.csv
- SupplementaryTable3allmodelsraw.csv
- SupplementaryTable1dataavailability.csv

Tables 1 to 5 are available in the Supplementary Files section.

## Figures and Tables

**Figure 2:**
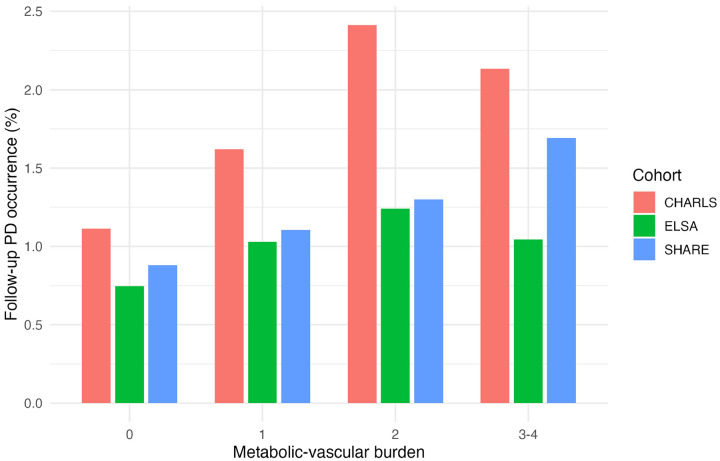
Follow-up reported Parkinson’s disease occurrence by metabolic-vascular burden. Bars show the percentage of participants with subsequent reported Parkinson’s disease within metabolic-vascular burden categories in CHARLS, ELSA, and SHARE. Burden was scored from 0 to 4 by summing diabetes, hypertension, dyslipidemia or high cholesterol, and BMI >=25 kg/m2; scores 3 and 4 are combined for descriptive display.

## Data Availability

This study used harmonised data from established ageing cohorts and harmonised data resources developed by the Gateway to Global Aging Data. Harmonised data files are distributed either by the originating study or through the Gateway, depending on the cohort. Access to source cohort and harmonised data is governed by the respective data-use policies and application procedures. Analysis outputs generated for this manuscript are available from the corresponding author upon reasonable request, subject to applicable data-use agreements and cohort-specific restrictions.

## References

[1] Kalia L. V. & Lang A. E. Parkinson’s disease. Lancet 386 (9996), 896–912. 10.1016/S0140-6736(14)61393-3 (2015). 25904081

[2] Schapira A. H. V., Chaudhuri K. R. & Jenner P. Non-motor features of Parkinson disease. Nat. Rev. Neurosci. 18 (7), 435–450. 10.1038/nrn.2017.62 (2017). 28592904

[3] Komici K., Femminella G. D., Bencivenga L., Rengo G. & Pagano G. Diabetes mellitus and Parkinson’s disease: a systematic review and meta-analyses. J. Parkinsons Dis. 11 (4), 1585–1596. 10.3233/JPD-212725 (2021). 34486987

[4] Chohan H. Type 2 diabetes as a determinant of Parkinson’s disease risk and progression. Mov. Disord. 36 (6), 1420–1429. 10.1002/mds.28551 (2021). 33682937 PMC9017318

[5] Nam G. E. Metabolic syndrome and risk of Parkinson disease: a nationwide cohort study. PLoS Med. 15 (8), e1002640. 10.1371/journal.pmed.1002640 (2018). 30130376 PMC6103502

[6] Ng Y. F. Case-control study of hypertension and Parkinson’s disease. npj Parkinsons Dis. 7, 63. 10.1038/s41531-021-00202-w (2021). 34290246 PMC8295270

[7] Chen J. Meta-analysis: overweight, obesity, and Parkinson’s disease. Int. J. Endocrinol. 2014, 203930. 10.1155/2014/203930 (2014). 24672544 PMC3941583

[8] Yue X. Risk of Parkinson disease in diabetes mellitus: an updated meta-analysis of population-based cohort studies. Med. (Baltim). 95 (18), e3549. 10.1097/MD.0000000000003549 (2016). PMC4863785 27149468

[9] Hou L. Hypertension and diagnosis of Parkinson’s disease: a meta-analysis of cohort studies. Front. Neurol. 9, 162. 10.3389/fneur.2018.00162 (2018). 29615961 PMC5867351

[10] Zhao Y., Hu Y., Smith J. P., Strauss J. & Yang G. Cohort profile: the China Health and Retirement Longitudinal Study (CHARLS). Int. J. Epidemiol. 43 (1), 61–68. 10.1093/ije/dys203 (2014). 23243115 PMC3937970

[11] Borsch-Supan A. Data resource profile: the Survey of Health, Ageing and Retirement in Europe (SHARE). Int. J. Epidemiol. 42 (4), 992–1001. 10.1093/ije/dyt088 (2013). 23778574 PMC3780997

[12] Steptoe A., Breeze E., Banks J. & Nazroo J. Cohort profile: the English Longitudinal Study of Ageing. Int. J. Epidemiol. 42 (6), 1640–1648. 10.1093/ije/dys168 (2013). 23143611 PMC3900867

[13] Lee J. Gateway to Global Aging Data: resources for cross-national comparisons of family, social environment, and healthy aging. J. Gerontol. B Psychol. Sci. Soc. Sci. 76 (Suppl 1), S5–S16. 10.1093/geronb/gbab050 (2021). 33861849 PMC8186854

[14] von Elm E. The STROBE Statement: guidelines for reporting observational studies. PLoS Med. 4 (10), e296. 10.1371/journal.pmed.0040296 (2007). 17941714 PMC2020495

[15] Bloem B. R., Okun M. S. & Klein C. Parkinson’s disease. Lancet 397 (10291), 2284–2303. 10.1016/S0140-6736(21)00218-X (2021). 33848468

[16] Dorsey E. R. Global, regional, and national burden of Parkinson’s disease, 1990–2016: a systematic analysis for the Global Burden of Disease Study 2016. Lancet Neurol. 17 (11), 939–953. 10.1016/S1474-4422(18)30295-3 (2018). 30287051 PMC6191528

[17] Ascherio A. & Schwarzschild M. A. The epidemiology of Parkinson’s disease: risk factors and prevention. Lancet Neurol. 15 (12), 1257–1272. 10.1016/S1474-4422(16)30230-7 (2016). 27751556

[18] de Lau L. M. L. & Breteler M. M. B. Epidemiology of Parkinson’s disease. Lancet Neurol. 5 (6), 525–535. 10.1016/S1474-4422(06)70471-9 (2006). 16713924

[19] Bellou V., Belbasis L., Tzoulaki I., Evangelou E. & Ioannidis J. P. A. Environmental risk factors and Parkinson’s disease: an umbrella review of meta-analyses. Parkinsonism Relat. Disord. 23, 1–9. 10.1016/j.parkreldis.2015.12.008 (2016). 26739246

[20] Santiago J. A. & Potashkin J. A. Shared dysregulated pathways lead to Parkinson’s disease and diabetes. Trends Mol. Med. 19 (3), 176–186. 10.1016/j.molmed.2013.01.002 (2013). 23375873

[21] De Pablo-Fernandez E., Goldacre R., Pakpoor J., Noyce A. J. & Warner T. T. Association between diabetes and subsequent Parkinson disease: a record-linkage cohort study. Neurology 91 (2), e139–e142. 10.1212/WNL.0000000000005771 (2018). 29898968

[22] Wang Y. L. Body mass index and risk of Parkinson’s disease: a dose-response meta-analysis of prospective studies. PLoS One. 10 (6), e0131778. 10.1371/journal.pone.0131778 (2015). 26121579 PMC4488297

[23] Huang X. Serum cholesterol and the progression of Parkinson’s disease: results from DATATOP. PLoS One. 6 (8), e22854. 10.1371/journal.pone.0022854 (2011). 21853051 PMC3154909

[24] Alberti K. G. M. M. Harmonizing the metabolic syndrome: a joint interim statement of. Circulation 120 (16), 1640–1645. 10.1161/CIRCULATIONAHA.109.192644 (2009). 19805654

[25] Postuma R. B. MDS clinical diagnostic criteria for Parkinson’s disease. Mov. Disord. 30 (12), 1591–1601. 10.1002/mds.26424 (2015). 26474316

[26] DerSimonian R. & Laird N. Meta-analysis in clinical trials. Control Clin. Trials. 7 (3), 177–188. 10.1016/0197-2456(86)90046-2 (1986). 3802833

